# Relationship between nutritional biomarkers and occlusal status in gastric cancer patients using the Eichner index: Observational study

**DOI:** 10.1097/MD.0000000000029094

**Published:** 2022-03-18

**Authors:** Atsushi Abe, Yu Ito, Hiroki Hayashi, Atsushi Nakayama, Hiroshi Furuta, Moeko Momokita, Hiroaki Hasegawa, Akari Tsunoda

**Affiliations:** *Department of Oral and Maxillofacial Surgery, Nagoya Ekisaikai Hospital, Nagoya, Japan.*

**Keywords:** occlusal status, postoperative infection, prognostic nutritional index, tooth loss

## Abstract

Systemic inflammatory responses and nutritional status are useful prognostic factors in gastric cancer patients. Since oral hypofunction causes undernutrition, we cross-sectionally investigated whether nutritional biomarkers were affected by the occlusal supporting zone status.

In 114 gastric cancer patients, the gastric cancer stage, body mass index, albumin levels, total lymphocyte counts, cholesterol levels, C-reactive protein levels, and 4 nutritional biomarkers - the Glasgow prognostic score (GPS), neutrophil-lymphocyte ratio, prognostic nutrition index (PNI), and controlling nutritional status (CONUT) - were evaluated. Oral conditions were assessed by determining the number of remaining teeth. The occlusal supporting status was based on the Eichner classification. Patients were assigned into 3 groups per their occlusal status, and mean values were compared using the Kruskal-Wallis test. The mean age and body mass index were 72.2 ± 8.5 (50-89) years and 22.0 ± 3.6 (14.8-33.4), respectively. There were 42, 39, 23, and 10 patients in stages I, II, III, and IV, respectively. The mean number of remaining teeth was 18.1 ± 9.5. According to the Eichner classification, there were 45, 42, and 27 patients in groups A, B, and C, respectively. The GPS and neutrophil-lymphocyte ratio values and CONUT frequencies between groups A and C were significantly different (*P* = .033, *P* = .00097, *P* = .04, respectively; Mann-Whitney *U* test). PNI values were lower in group C with poor occlusal support zones than in group A with stable occlusal support zones.

Occlusal supporting zone reductions were undernutrition associated. Eichner Class C patients with few occlusal supporting zones had poor GPS, PNI, and CONUT values and were undernourished.

## 1. Introduction

Gastric cancer is one of the most lethal forms of cancer worldwide, with an estimated 951,600 new cases and 723,100 deaths in 2012.^[1,2]^ Despite early detection, improvements in surgical procedures, and availability of advanced chemotherapeutic options, long-term survival is still unsatisfactory due to the associated local recurrence or distant metastasis. The immune and nutritional statuses of patients have been reported to be associated with postoperative outcomes in malignant tumors.^[3-6]^ The nutritional status of patients with cancer is often evaluated by physical examination and blood biochemical findings such as albumin (ALB).^[7]^ Patients with gastric cancer who are immunocompromised due to pre-operative malnutrition or systemic inflammatory responses often have delayed wound healing, develop complications, and have increased mortality rates.^[8-12]^ The nutritional status and systemic inflammatory responses in patients with cancer are considered to be useful prognostic factors.^[13-15]^ Therefore, various biomarkers that reflect the inflammatory state, nutritional status, and immunocompetence of patients have been reported as valuable prognostic factors.^[16-20]^

Pre-operative undernutrition is due to multiple biological, physiological, and pathological factors, one of which is decreased oral function. Oral dysfunction, which causes undernutrition, includes impaired masticatory ability due to tooth loss, dry oral mucosa, and infection. Such conditions affect food choices and result in a reduced quality of life, development of severe complications, and increased mortality rates. A change in food selection is often observed with a decline in masticatory efficiency due to tooth loss and malocclusion.^[21]^ People with chewing difficulties eventually consume less nutritious foods such as vegetables, fruits, meats, and grains.^[21-23]^ Hence, a decrease in oral cavity function results in a poor pre-operative nutritional status during gastric cancer surgery, with the possibility of an increase in the occurrence of postoperative complications.

Although there have been studies that investigated how a decrease in the number of teeth and impaired masticatory function affect nutritional status, studies that determine the association between nutritional status before gastrointestinal cancer surgery and oral conditions of patients are few. Currently, it is uncertain whether the intraoral state of the patient affects either blood cells or plasma components, and to determine these effects would require a novel evaluation approach. In the present cross-sectional study, we investigated whether some biomarkers (Glasgow prognostic score [GPS], neutrophil-lymphocyte ratio [NLR], controlling nutritional status [CONUT], and prognostic nutrition index [PNI]), pre-operative nutritional markers in patients with gastric cancer, would differ according to the occlusal support zone status. From this assessment, we determined how the occlusion support zone status is associated with various biomarkers in the blood.

## 2. Methods

### 2.1. Subjects

This cross-sectional study enrolled 145 patients who were pathologically diagnosed with primary gastric cancer and underwent a dental examination before surgery at the Nagoya Ekisaikai Hospital between September 2014 and March 2019. Of these patients, 31 who had recurrent cancer, metabolic diseases (e.g., diabetes mellitus), or incomplete data were excluded. In the present study, 114 patients (85 men and 29 women) were included.

### 2.2. Assessment of gastric cancer and general conditions

Gastric cancer was diagnosed by consensus among skilled physicians. While assessing gastric cancer, the onset site and cancer stage was determined based on the results of endoscopy, computed tomography, and magnetic resonance imaging performed at the department of gastrointestinal surgery. The body mass index (BMI) of each patient was also calculated. Blood samples were collected on admission, during the pre-surgical examination (within 1 month before surgery). From these blood samples, ALB levels, total lymphocyte counts, and C-reactive protein (CRP) levels were measured. Based on these pre-operative blood test results, the severity of inflammation was assessed.

### 2.3. Evaluation of nutritional biomarker

We evaluated 4 kinds of nutritional biomarkers. The biomarkers evaluated were GPS,^[24,25]^ NLR,^[26,27]^ CONUT,^[28,29]^ and PNI,^[17,18]^ as described in Tables 1 and 2. The GPS was calculated using pre-operative CRP and ALB values. Patients with raised concentrations of CRP (over 10mg/L) and hypoalbuminemia (ALB < 35 g/L) were given a score of 2. Those with raised concentrations of CRP without hypoalbuminemia were given a score of 1. Patients who did not have raised concentrations of CRP were given a score of 0 regardless of their ALB status. NLRs were also calculated. A NLR of ≥5 was given a score of 1; if it was < 5, the score was 0. PNI was calculated on the basis of serum ALB levels and peripheral blood lymphocyte count using the following formula.

**Table 1:** Calculation of the Glasgow prognostic score (GPS).

**Classification**	**Points**
C-reactive protein ≦ 1.0 mg/dL and albumin ≧ 3.5 g/dL	0
C-reactive protein > 1.0 mg/dL or albumin < 3.5 g/dL	1
C-reactive protein > 1.0 mg/dL and albumin < 3.6 g/dL	2

**Table 2:** Calculation of the CONUT score.

**Parameter**	**None**	**Light**	**Moderate**	**Severe**
Serum albumin, g/dL	≥3.50	3.00-3.49	2.50-2.99	<2.50
Score	0	2	4	6
Total lymphocyte, count, /mm^3^	≥1600	1200-1599	800-1199	<800
Score	0	1	2	3
Total cholesterol, mg/dL	≥180	140-179	100-139	<100
Score	0	1	2	3
CONUT = controlling nutritional status.				

PNI = [10 × serum ALB level (g/dL)] + [0.005 × total peripheral lymphocyte count (per mm^3^)]

The serum ALB concentration, total peripheral lymphocyte count, and total cholesterol concentration were used to obtain the CONUT score. The scores were added up and patients were categorized into the following groups based on the cumulative score: normal (0-1), mild disorder (2-4), moderate disability (5-8), and severe disability (>8).

### 2.4. Assessment of oral conditions

Oral conditions were assessed by 2 dentists who were blinded to patients’ nutritional status in the present study. In the oral examination, the remaining teeth excluding residual dental roots were counted, and the occlusal support zone status was assessed according to the Eichner classification.^[7]^ The Eichner classification is a method used to evaluate occlusion support zone status based on the remaining teeth. The zone is defined as a site supporting occlusion. In a healthy dentition composed of opposing tooth pairs in the right and left premolar and molar regions, occlusion is supported by 4 occlusal supporting zones. The Eichner index categorizes the occlusal status into 3 main groups (A, B, and C). The number of remaining teeth and Eichner classification are so objective that they can be regarded as very reliable.

In patients using dentures, their dentures were examined by a dentist. The occlusal supporting zones were assessed for the contact between opposite tooth pairs.

### 2.5. Statistical analyses

The number of cases during the study period determined the sample size. We also examined whether the values of the 4 biomarkers differed among the 3 Eichner Classes A, B, and C. We considered whether there was a difference in GPS and CONUT frequencies between the 3 Eichner groups. Categorical data (GPS and CONUT) were analyzed using χ^2^ tests, except when expected cells were found to be less than 5, in which case, Fisher exact test was employed. The Shapiro-Wilk test was used to assess the distribution of pre-operative NLR and PNI values, and the normality was confirmed at a 5% significance level. Because NLR and PNI were not normally distributed (*P* = .000003493 and .02542), the 3 groups were compared using the Kruskal-Wallis test, which is a non-parametric test. In addition, Steel-Dwass multiple comparison tests were performed to determine which of the 3 groups showed differences in NLR and PNI. All statistical analyses were performed with EZR (Saitama Medical Center, Jichi Medical University, Saitama, Japan), which is a graphical user interface for R (The R Foundation for Statistical Computing, Vienna, Austria). More precisely, it is a modified version of R commander designed to add statistical functions frequently used in biostatistics.^[24]^ Based on the effect size observed in our study, the estimated sample size was 44 patients per group for a total of 88 patients. Sample size estimates were based on the following: standard deviation within groups 5.0, alevel, 0.5 and power of 80%.

### 2.6. Ethics

This cross-sectional study was approved by the Nagoya Ekisaikai Hospital Ethics Committee (approval number 2017-040). The study was conducted in accordance with the Strengthening the Reporting of Observational Studies in Epidemiology (STROBE) statement guidelines for reporting observational studies and the Declaration of Helsinki. Informed consent was obtained from the patient for the purpose of publication.

## 3. Results

### 3.1. Patient characteristics

None of the markers of systemic and oral conditions was normally distributed. Patients were classified into 3 groups according to the occlusal support zone status based on the Eichner classification to compare each marker. Table 1 shows the patient characteristics. The mean age of the patients was 72.2 ± 8.5 years (range, 50-89 years). The mean BMI was 22.0 ± 3.5 (range, 14.8-33.4). There were 42, 39, 23, and 10 stages I, II, III, and IV patients, respectively. According to the Eichner classification, there were 45, 42, and 27 patients in groups A, B, and C, respectively. No significant differences were observed in sex, age, gastric cancer stage, BMI, or CRP levels among the 3 Eichner Classes A, B, and C. Concerning denture usage, 45 of the 143 patients used dentures, and 69 did not. Among these patients using dentures, the number of those without occlusal supports was 4, 22, and 19 in groups A, B, and C, respectively (Table 3).

**Table 3:** Patient characteristics.

**Factor**	**Group**	**Overall**
n		114
Denture (0: yes, 1: no; %)	0	45 (39.5)
	1	69 (60.5)
Eichner.1.A.2.B.3.C (%)	1	45 (39.5)
	2	42 (36.8)
	3	27 (23.7)
Sex (0: male, 1: female; %)	0	85 (74.6)
	1	29 (25.4)
Stage (%)	1	41 (36.0)
	2	39 (34.2)
	3	25 (21.9)
	4	9 (7.9)
Age		71.83 (10.00)
ALB		3.71 (0.57)
BMI		21.99 (3.57)
CRP		0.67 (1.54)
Lymph		26.09 (8.22)
Neutrophil		65.07 (8.91)
NLR		3.14 (2.78)
PNI		40.42 (6.25)
Remaining teeth		18.10 (9.54)
Total lymph		1643.47 (851.77)
WBC		6.94 (8.60)
ALB = albumin, BMI = body mass index, CRP = C-reactive protein, NLR = neutrophil-lymphocyte ratio, PNI = prognostic nutrition index, WBC = white blood cells.		

### 3.2. Oral conditions

The mean number of remaining teeth was 18.1 ± 9.5 (range, 0-31 teeth). Even among patients with the same number of remaining teeth, if the number of occlusal supporting zones differs, their nutritional status may be differently affected. Thus, differences in nutritional biomarkers were analyzed according to the Eichner classification. The nutritional biomarkers are shown in Table 2 according to Eichner Classes A, B, and C. The CRP levels were 0.5 ± 1.1, 0.5 ± 0.7, and 1.7 ± 3.3 in groups A, B, and C, respectively (*P* = .01), indicating significant differences among the 3 groups. The ALB levels were 3.9 ± 0.45, 3.68 ± 0.5, and 3.43 ± 0.7 in groups A, B, and C, respectively (*P* = .002), indicating significant differences among the 3 groups (Table 4).

**Table 4:** Patient characteristics of Eichner classification.

		**Eichner**
**Factor**	**Group**	**A**	**B**	**C**	***P* value**
n		45	42	27
Denture (%)	Yes	4 (8.9)	22 (52.4)	19 (70.4)	< .001
	No	41 (91.1)	20 (47.6)	8 (29.6)
Sex (%)	Male	32 (71.1)	33 (78.6)	20 (74.1)	.725
	Female	13 (28.9)	9 (21.4)	7 (25.9)
Stage (%)	1	16 (35.6)	13 (31.0)	12 (44.4)	.734
	2	16 (35.6)	13 (31.0)	10 (37.0)
	3	10 (22.2)	12 (28.6)	3 (11.1)
	4	3 (6.7)	4 (9.5)	2 (7.4)
Age		68.51 (10.81)	74.21 (8.86)	73.67 (9.00)	.015
ALB		3.91 (0.45)	3.68 (0.51)	3.43 (0.71)	.002
BMI		21.98 (3.23)	22.69 (3.53)	20.94 (4.04)	.137
CRP		0.47 (1.09)	0.49 (0.78)	1.26 (2.61)	.07
Lymph		27.44 (7.99)	26.04 (7.84)	23.92 (8.98)	.214
Neutrophil		63.89 (8.70)	64.80 (7.88)	67.46 (10.53)	.251
NLR		2.66 (1.26)	2.97 (1.92)	4.22 (4.84)	.061
PNI		42.49 (4.87)	40.34 (5.81)	37.11 (7.59)	.001
Total lymph		1687.57 (592.20)	1760.62 (1152.40)	1387.72 (609.94)	.188
WBC		6.24 (1.65)	8.34 (14.00)	5.93 (1.74)	.412
ALB = albumin, BMI = body mass index, CRP = C-reactive protein, NLR = neutrophil-lymphocyte ratio, PNI = prognostic nutrition index, WBC = white blood cells.					

GPS is a common score used to assess levels of inflammation. The score assessments revealed frequencies of 32 GPS level 0, 4 GPS level 1, 5 GPS level 2, and 4 GPS level 3 in group A, 21 GPS level 0, 9 GPS level 1, 6 GPS level 2, and 6 GPS level 3 in group B, and 11 GPS level 0, 2 GPS level 1, 3 GPS level 2, and 11 GPS level 3 in group C (*P* = .0179), indicating significant differences among the 3 groups. In addition, multiple comparisons revealed no difference in GPS levels between groups A and B, and between groups B and C (*P* = .615, *P* = .23), but significant differences were observed between groups A and C (*P* = .033) (Mann-Whitney *U* test) (Fig. 1).

**Figure:**
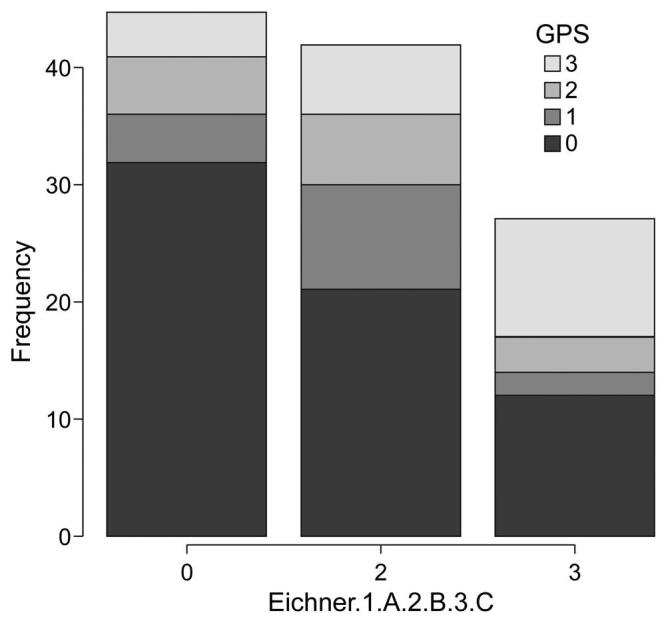
**Figure 1.** Comparison of GPS levels among the 3 groups based on the Eichner index. GPS = Glasgow prognostic score.

The NLR (nutrient rating system using blood cells) values were 2.7 ± 1.3, 3.0 ± 1.9, and 4.2 ± 4.8 in groups A, B, and C, respectively (*P* = .217), indicating no significant difference among the 3 groups (Fig. 2).

**Figure:**
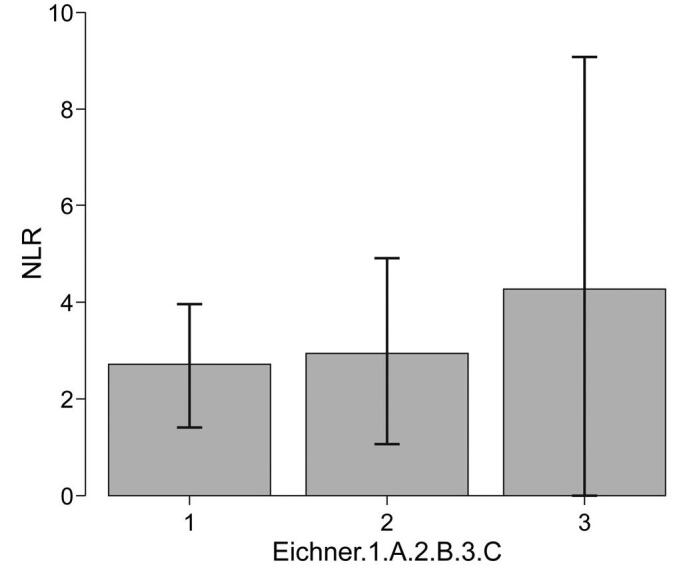
**Figure 2.** Comparison of NLR levels among the 3 groups based on the Eichner index. NLR = neutrophil-lymphocyte ratio.

The PNI is calculated based on the serum ALB concentration and peripheral blood lymphocyte count. The values for patients in this study were 42.6 ± 4.9, 40.3 ± 5.8, and 37.1 ± 7.6 in groups A, B, and C, respectively (*P* = .0048), indicating significant differences among the 3 groups. In addition, multiple comparisons revealed no difference in either ALB levels or PNI between groups A and B, and between groups B and C (*P* = .29, *P* = .06), but significant differences were observed between groups A and C (*P* = .00097) (Mann-Whitney *U* test). These results revealed that PNI values were lower in group C with poor occlusal support than in group A with stable occlusal support (Fig. 3).

**Figure:**
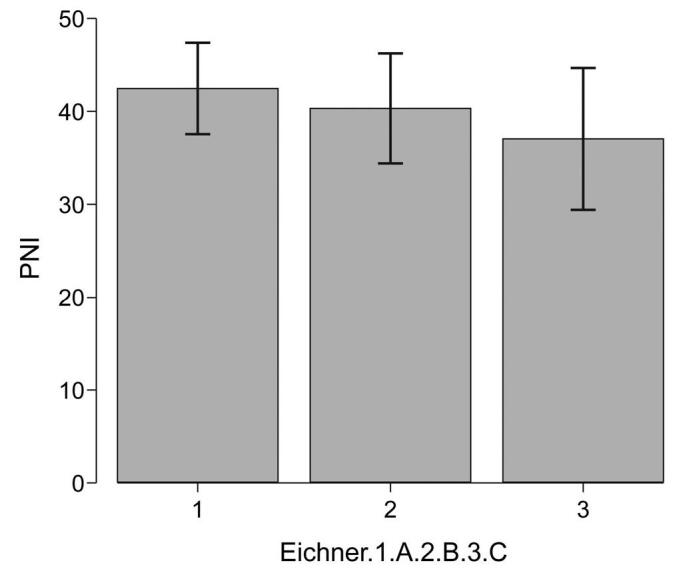
**Figure 3.** Comparison of PNI levels among the 3 groups based on the Eichner index. PNI = prognostic nutrition index.

The CONUT score is calculated according to serum ALB concentration, total peripheral lymphocyte count, and total cholesterol concentration. The score frequencies for this assessment were 25 CONUT level 1, 15 CONUT level 2, 4 CONUT level 3, and 1 CONUT level 4 in group A, 15 CONUT level 1, 16 CONUT level 2, 10 CONUT level 3, and 1 CONUT level 4 in group B, and 6 CONUT level 1, 12 CONUT level 2, 5 CONUT level 3, and 4 CONUT level 4 in group C (*P* = .03), indicating significant differences among the 3 groups. In addition, multiple comparisons revealed no difference in GPS levels between groups A and B, and between groups B and C (*P* = .39, *P* = .6), but significant differences were observed between groups A and C (*P* = .04) (Mann-Whitney *U* test) (Fig. 4).

**Figure:**
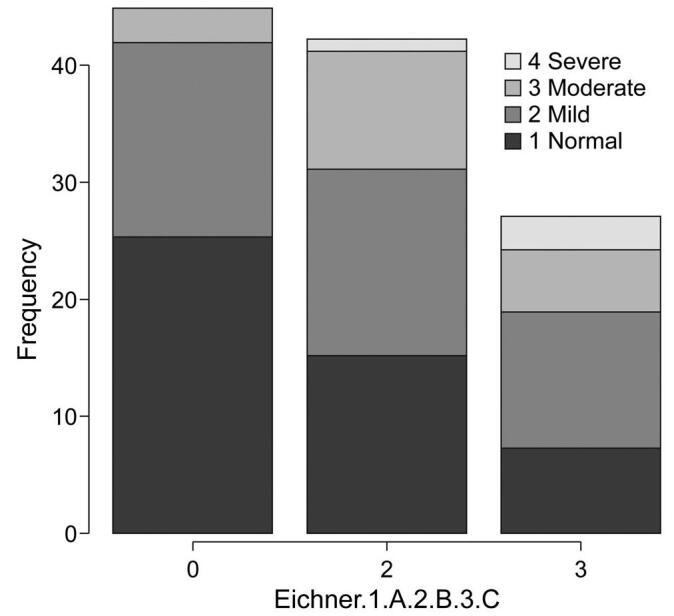
**Figure 4.** Comparison of CONUT levels among the 3 groups based on the Eichner index. CONUT = controlling nutritional status.

Thus, the present study revealed that GPS, PNI, and CONUT were affected by the numbers of remaining teeth and occlusal supporting zones.

## 4. Discussion

The oral cavity is an important organ for nutrient intake, and the nutritional status is affected by impaired oral function due to the hypofunction of the temporomandibular joints, masticatory muscles, missing teeth, and impaired swallowing function.^[30-32]^ In the present study, the examination of the occlusal support zone status and nutritional biomarkers revealed significant differences in the GPS and CONUT levels and PNI values. The Eichner C group without occlusal support had higher GPS levels than did group A, and the CONUT levels revealed moderate or severe malnutrition; the PNI values were low in this group. Therefore, decreased occlusal support was associated with GPS, CONUT, and PNI, and was considered to be one of the factors of malnutrition. However, there was no significant difference in NLR between the 3 groups. Furthermore, nutritional evaluations using the protein levels of the blood revealed significant differences in the oral cavity function failure among the 3 groups; whereas, a significant difference was not found in nutritional evaluation using the blood cells. Therefore, the change in diet due to oral cavity function disorder reduced plasma proteins such as ALB and cholesterol and was thought to result in undernutrition and poor immunocompetence. The results suggested that impaired oral function due to oral diseases and tooth loss reduces dietary intake and is associated with undernutrition. The correlation between the number of residual teeth and nutritional status has been reported in many studies, and the results of the present study are comparable with those of such studies. Meanwhile, the bite force varies depending on periodontal disease and stability of the remaining teeth, and it affects the food choice and nutritional status. Thus, even among patients with the same number of remaining teeth, their conditions may differ depending on the sites and arrangement of the remaining teeth. Although the Eichner classification is based on the assessment of the occlusal support zone status at the time of clinical examination, it may help make a diagnosis of oral frailty^[33]^ after a certain period because the simultaneous loss of multiple teeth rarely occurs.

Eichner A has all occlusal support areas; Eichner B is a case of reduced occlusal support areas. Eichner C has no occlusal support areas. When the occlusal support of the molars is stabilized, patients can consume more proteins such as meat and fish, and dietary fibers such as vegetables and fruits. On the other hand, patients with less occlusal support between upper and lower teeth are more likely to avoid tough, fibrous, and dry foods and engage in inappropriate eating habits.^[34,35]^ Eichner classification B/C have lower masticatory ability than Eichner classification A, which affects their eating habits. Eichner classification is associated with nutritioned status.

In patients with poor pre-operative nutritional status, the relative risks of postoperative complications and in-hospital death are considered to be increased by 2 to 4 folds. Cell-mediated immunity is most affected by undernutrition. Undernourished people exhibit atrophy of the thymus, reduced peripheral T cell counts, reduced CD4/CD8 ratios (CD4 dominant), and atrophy of lymph nodes, spleen, and gut-associated lymphoid tissue, which is the immune tissue of the intestinal tract. Atrophy of gut-associated lymphoid tissue reduces the immunity of the intestinal mucosa and causes gastrointestinal infection. In addition, severe malnutrition reduces immunoglobulin levels. On the other hand, neither neutrophil nor peripheral B lymphocyte count decreases, but the phagocytic capacity, bactericidal capacity, and complement production are reduced.^[36-39]^ Biomarkers for assessing systemic inflammatory responses and the nutritional status include the GPS,^[24,25]^ neutrophil/lymphocyte ratio,^[26,27]^ lymphocyte/monocyte ratio,^[33,40]^ and CRP/ALB ratio.^[41]^ These markers are calculated based on serum protein levels (e.g., CRP and ALB), cell counts (e.g., neutrophil, lymphocyte, and monocyte), and ratios of these values. Each nutrient rating system has its intrinsic characteristics. There are limitations to the use of a single index; thus, we chose appropriate nutritional indexes and evaluated them based on the disease stage and the therapeutic option employed.

The GPS was the index reported in non-small lung cell cancer by Macmillan, and the GPS combines CRP and ALB and evaluates blood protein moieties primarily. The GPS has been reported in various cancer studies to be a prognostic marker. The poor prognosis case shows systemic inflammatory such as the GPS Category 2 and 3 and undernutrition. The CRP level of patients with cancer reflects IL-6 levels in the blood, and a persistent increase in IL-6 levels suggests inflammation of the carcinoma tissue.^[42]^ Whereas, the progression of periodontal disease and the presence of dental caries cause odontogenic infectious diseases and an increase in CRP. However, between the 3 Eichner groups, a significant difference was not found in CRP levels. The effect on GPS had little impact on teeth and chronic odontogenic infectious diseases in this examination, while for effect on GPS category, the ALB was thought to be more important. Systemic inflammatory responses are assessed as changes in biochemical data that result from activation of the immune system by the presence of tumors and secretion of IL-6 and other proinflammatory cytokines.^[42-44]^

NLR is a nutrient rating system using blood cells (neutrophils and lymphocytes). Lymphocytes have been used as one of the nutritional evaluation indexes for a long time.^[45]^ Lymphocytes are used as an indicator of immunity. The lymphocytes are related to tumor immunity and act for a tumor restrainingly.^[46,47]^ Whereas neutrophils increase due to inflammation and induce the production of chemokines and cytokine, the produced chemokines and cytokines enhance the growth, invasion, and neovascularization of tumors. Therefore, tumor growth is closely associated with inflammation.^[48,49]^ NLR, which evaluates CRP and ALB ratios, is reported as an oncological prognostic marker similar to GPS. A significant difference was not found in this examination between the 3 Eichner groups. NLR is considered to mirror a balance between the innate and adaptive immune mechanisms.^[49]^ The NLR can reflect the initial innate immune mechanisms (involving cells such as neutrophils and macrophages that provide a non-specific response), which triggers the adaptive immune mechanisms (T-cell/B-cell mediated, and partly PLT stimulated) that result in periodontal destruction. In cases of high NLR values, periodontitis becomes more severe.^[47]^ It is reported that NLR is useful for clinical evaluations of periodontitis. A mouth cleaning state is good in Eichiner A where many residual teeth are present in, and the inflammatory reaction is poor. Additionally, there is no effect as we raise CRP because Eichner C has few residual teeth causing odontogenic infectious diseases. The difference was not found in such a situation. Whereas there are many patients that a lot of residual teeth have a good mouth cleaning state in Eichner A. Also, Eichner C has few residual teeth and does not reach it before we greatly change CRP even if periodontal disease is severe because there are few teeth causing inflammation. A difference was not found in such a situation. PNI and CONUT are indexes that are dependent on the evaluation of lymphocyte counts, ALB, and cholesterol. It has been reported that it has a poor prognosis in a low level of PNI and the extensive malnutrition category of CONUT. The lymphocytes have antitumor immunity eligibility. The lymphocytes commit a tumor restrainingly and have antitumor immunocompetence.^[48]^ The half-life in the blood of cholesterol 8 days, shorter than 21 days for ALB, and reflects malnutrition subtly.^[21-23,50]^ Significant differences were found between the 3 groups and PNI and CONUT proved to the more reflective of undernutrition in group C than in group A. Wakai et al^[50]^ reported that as the number of teeth decreases, people consume fewer vegetables but their total calorie intake increases with an increase in the consumption of carbohydrate such as rice, and other confectionaries. If the occlusion status is poor, carbohydrates and glucose play key dietary roles. Simultaneously, there is a reduced intake of neutral fat or proteins. As a result, these indexes are thought to worsen.

The present study has several limitations. Nutritional status is affected by many factors. This study was conducted as a retrospective, exploratory observational study of single center. There are many complex factors that influence the nutritional status of occlusion. In order to better understand its manifestation, more cases need to be accumulated. It is difficult to recognize the causes of changes in masticatory function in the elderly. These changes may be caused by aging, sensory or physiological changes, disease, or drug-related processes. The biomarkers examined in this study are calculated based on the measurement of ALB levels and blood cell counts. They are decreased by dehydration, increased vascular permeability associated with vascular endothelial damage and systemic inflammation, and liver dysfunction. Chemotherapy can also affect nutritional biomarkers by decreasing ALB levels and blood cell counts associated with drug toxicity. The biomarkers are likely to be frequently variable, when patients do pre-operative chemotherapy or those with severe inflammation or autoimmune diseases. We should be consider carefully about the patients. In addition to oral findings such as prosthodontic treatment with dentures and conditions of the remaining teeth, other factors including socioeconomic strata, income levels, and acquired behavioral patterns are also very important.^[47]^ It was impossible to control all of these potential variables in the present study. Furthermore, to determine whether dentures functioned well, factors that can influence outcomes such as the levels of technical skills of dentists, designs of dentures, the difficulty of cases, skills of dental technicians who fabricate dentures, perfection levels of dentures, and the actual use of dentures, should be assessed. However, there are no standardized methods to assess these factors, and only a few studies have reported the evaluation of these factors. These factors should be assessed in future studies. Because the present study is a cross-sectional study, no causal relationship could be determined. Thus, we cannot state that the number of teeth or occlusal status directly affects nutritional status. Our results are based on the experience with a small number of patients at single institution. Therefore, the generality of our findings are proven for more further studies.

An early transition to oral intake is essential in the treatment of gastric cancer. The reasons for this are not only that oral intake is the best enteral nutrition, but also that eating through the mouth is an important activity, which is associated with human dignity and affects quality of life. In addition to the problems of undernutrition, prolonged lack of oral intake leads to a disuse hypofunction of the eating and swallowing muscles. Thus, a delayed start of oral intake due to complications, such as postoperative ruptured suture, should be avoided, and efforts should be made to precisely diagnose and improve pre-operative undernutrition. Given the importance of the function of the remaining teeth for nutrient intake and the unduly significant effects of impaired masticatory function on nutritional status, dentists and dental hygienists in a nutrition support team play an important role in maintaining and improving oral function, which is important for nutrient intake. For example, the oral cavity should be kept clean, and the periodontal condition should be maintained. In addition, patients with occlusal disharmony may be undernourished, as an underlying condition, even if they are satisfied with denture use. Thus, occlusal disharmony is suggested to be a possible factor for the consideration of preoperative nutritional supplementation. Based on this study, there are 2 conclusions that can be drawn regarding malnutrition and inflammation in patients with gastric cancer patients with oral health conditions: There are direct effects of occlusal abnormalities on dietary intake, and there is indirect action of systemic inflammatory conditions such as odontogenic infectious diseases on acute protein synthesis.

Further studies on oral health and its role in malnutrition and inflammation are warranted. A long-term prospective study and intervention study are necessary to completely evaluate the association among systemic inflammation in oral health, malnutrition, and gastric cancer. Patients with gastric cancer who are eligible for surgery need to undergo a variety of screening tests. One of them includes dental evaluation. Pre-operative dental examinations are one of the most important tests to assess nutritional status. The pre-operative oral evaluation should assess the occlusal status in addition to the oral cleaning status, presence of moving teeth, and prevent postoperative pneumonia. For patients with poor occlusion, it is necessary to re-evaluate nutritional management, consult with nurses, physicians in charge, and dietitians, and if necessary, consider requesting Nutrition Support Team intervention.

## 5. Conclusions

The association between the nutritional biomarkers and dental conditions of patients with gastric cancer with different occlusal statuses was examined. Decreases in the occlusal supporting zones were correlated with undernutrition. Patients with fewer occlusal supporting zones had lower ALB levels and PNI, and GPS and CONUT levels were found to be reduced in undernourished patients. The Eichner C group was inferior to other groups in GPS, PNI, and CONUT. The occlusion status affects humoral ingredients such as ALB. This study emphasizes an important role of oral health in improving malnutrition in gastric cancer. Furthermore, it shows that it predicts the decrease of a nutrient in gastric cancer and the caloric intake that the oral cavity appearing by the loss of teeth is in bad health. Furthermore, oral functional decline due to the tooth missing shows that we predict a decrease in the nutrition of patients with gastric cancer and systemic inflammatory reaction.

## Acknowledgments

We would like to thank Editage Science Communications for English language editing and publication support.

## Author contributions

**Conceptualization:** Atsushi Abe.

**Data curation:** Moeko Momokita.

**Formal analysis:** Hiroaki Hsegawa.

**Investigation:** Akari Tsunoda.

**Methodology:** Atsushi Abe, Atsushi Nakayama.

**Project administration:** Atsushi Abe.

**Resources:** Hiroki Hayashi, Atsushi Nakayama.

**Supervision:** Hiroshi Furuta.

**Validation:** Yu Ito.

**Visualization:** Hiroaki Hsegawa.

**Writing - original draft:** Atsushi Abe.

**Writing - review & editing:** Atsushi Abe.
